# Complete Genome Sequence of the Bioluminescent Marine Bacterium Vibrio harveyi ATCC 33843 (392 [MAV])

**DOI:** 10.1128/genomeA.01493-14

**Published:** 2015-01-29

**Authors:** Zheng Wang, W. Judson Hervey, Seongwon Kim, Baochuan Lin, Gary J. Vora

**Affiliations:** a Center for Bio/Molecular Science and Engineering, U.S. Naval Research Laboratory, Washington, DC, USA; b National Academy of Sciences, National Research Council, U.S. Naval Research Laboratory, Washington, DC, USA

## Abstract

Vibrio harveyi is a Gram-negative marine γ-proteobacterium that is known to be a formidable pathogen of aquatic animals and is a model organism for the study of bacterial bioluminescence and quorum sensing. In this report, we describe the complete genome sequence of the most studied strain of this species: V. harveyi ATCC 33843 (392 [MAV]).

## GENOME ANNOUNCEMENT

Vibrio harveyi is central member of the Harveyi clade (1) and Vibrio core group (2, 3) that is primarily found in tropical and temperate marine environments as a free-living organism or in association with eukaryotes as a commensal, opportunistic pathogen, or primary pathogen (4–8). The study of this bacterium has a rich yet convoluted history filled with several changes in classification and nomenclature (originally named Achromobacter harveyi (9); junior synonyms Vibrio trachuri (10), Vibrio carchariae (11); basonym Lucibacterium harveyi; other synonyms Beneckea neptuna, Beneckea harveyi, Pseudomonas harveyi, Photobacterium harveyi), incorrectly identified strains (6, 12–14), and seminal scientific contributions. Perhaps most importantly, V. harveyi has played a principal role in our understanding of the genetics and biochemistry of bacterial bioluminescence (15, 16) and was one of two species in which autoinduction was first described (17). One strain in particular, V. harveyi ATCC 33843 [chain of custody—J. W. Hastings (MAV) → P. Baumann (392) → ATCC (18); various strain identifiers MAV, 392, B-392, CAIM 520, LMG 11226, NCCB 79042], has served as a model system in the discovery and understanding of autoinduction (17), alloinduction (19), autoinducer structure (20), and transcriptional regulation of bioluminescence (21, 22).

While interest in this organism remains high, documented misidentifications (12, 13) have confounded the conventional knowledge regarding V. harveyi and its sister species and have resulted in the current absence of a fully sequenced V. harveyi genome. To correct this oversight, we sequenced the genome of V. harveyi ATCC 33843 using the Pacific Biosciences *RS* II sequencing platform (DNA Link USA, Inc., San Diego, CA). Briefly, genomic DNA was extracted using the Gentra Puregene yeast/bacteria kit (Qiagen, Valencia, CA) and used to prepare a 10-kb insert library that was sequenced using two single-molecule real-time (SMRT) sequencing cells and P4-C2 chemistry. This resulted in 31,919 filtered and preassembled sequence reads with a mean length of 4,290 bp and 23× genome coverage. Assembly (via SMRTpipe HGAP.2 and SMRTpipe Celera Assembler) and consensus polishing (SMRTpipe Quiver) yielded two circular chromosomes (3,621,606-bp Chr I and 2,259,884-bp Chr II) with a finished genome size of 5,881,490 bp (44.9% GC content). Gene prediction and annotation were performed using GeneMarkS+ and the NCBI Prokaryotic Genome Annotation Pipeline, respectively, and identified 5,133 coding sequences, of which 825 were predicted to encode hypothetical proteins.

The genome was found to contain a 97.5-kbp superintegron, 3 integrated prophages or phage remnants, 12 rRNA operons, 7 transposases, and 0 retrons. Also found were genes encoding the type II, III, IV, and VI secretion systems, lateral and polar flagellar systems, polyhydroxybutyrate synthesis (23), bioluminescence (*luxCDABEGH*), and the full complement of established quorum-sensing-associated proteins that have been most extensively described in Vibrio campbellii strain BAA-1116 (13, 24). This effort provides a necessary foundation to further understand the gene regulation and phenotypes that have been ascribed to this strain over the course of more than four decades.

### Nucleotide sequence accession numbers.

This whole-genome project has been deposited at DDBJ/EMBL/GenBank under the accession numbers CP009467.2 and CP009468.1.
